# Raman and autofluorescence spectroscopy for in situ identification of neoplastic tissue during surgical treatment of brain tumors

**DOI:** 10.1007/s11060-024-04809-w

**Published:** 2024-08-28

**Authors:** Ortrud Uckermann, Jonathan Ziegler, Matthias Meinhardt, Sven Richter, Gabriele Schackert, Ilker Y. Eyüpoglu, Mido M. Hijazi, Dietmar Krex, Tareq A. Juratli, Stephan B. Sobottka, Roberta Galli

**Affiliations:** 1 Division of Medical Biology, Department of Psychiatry and Psychotherapy, Faculty of Medicine, University Hospital Carl Gustav Carus, Technische Universität Dresden, Dresden, Germany; 2 Department of Neurosurgery, Faculty of Medicine and University Hospital Carl Gustav Carus, Technische Universität Dresden, Dresden, Germany; 3 Medical Physics and Biomedical Engineering, Faculty of Medicine, Technische Universität Dresden, Dresden, Germany; 4 Department of Pathology (Neuropathology), Faculty of Medicine and University Hospital Carl Gustav Carus, Technische Universität Dresden, Dresden, Germany; 5 Else Kröner Fresenius Center for Digital Health, Faculty of Medicine, Technische Universität Dresden, Dresden, Germany

**Keywords:** Glial tumors, Brain metastases, Raman spectroscopy, Autofluorescence, Intraoperative

## Abstract

**Purpose:**

Raman spectroscopy (RS) is a promising method for brain tumor detection. Near-infrared autofluorescence (AF) acquired during RS provides additional useful information for tumor identification and was investigated in comparison with RS for delineating brain tumors in situ.

**Methods:**

Raman spectra were acquired together with AF in situ within the solid tumor and at the tumor border during routine brain tumor surgeries (218 spectra; glioma WHO II-III, *n* = 6; GBM, *n* = 10; metastases, *n* = 10; meningioma, *n* = 3). Tissue classification for tumor identification in situ was trained on ex vivo data (375 spectra; glioma/GBM patients, *n* = 20; metastases, *n* = 11; meningioma, *n* = 13; and epileptic hippocampi, *n* = 4).

**Results:**

Both in situ and ex vivo data showed that AF intensity in brain tumors was lower than that in border regions and normal brain tissue. Moreover, a positive correlation was observed between the AF intensity and the intensity of the Raman band corresponding to lipids at 1437 cm^− 1^, while a negative correlation was found with the intensity of the protein band at 1260 cm^− 1^. The classification of in situ AF and RS datasets matched the surgeon’s evaluation of tissue type, with correct rates of 0.83 and 0.84, respectively. Similar correct rates were achieved in comparison to histopathology of tissue biopsies resected in selected measurement positions (AF: 0.80, RS: 0.83).

**Conclusions:**

Spectroscopy was successfully integrated into existing neurosurgical workflows, and in situ spectroscopic data could be classified based on ex vivo data. RS confirmed its ability to detect brain tumors, while AF emerged as a competitive method for intraoperative tumor delineation.

**Supplementary Information:**

The online version contains supplementary material available at 10.1007/s11060-024-04809-w.

## Introduction

In the surgical management of malignant brain tumors, achieving maximal resection improves progression-free and overall survival. Various techniques, such as neuronavigation [1, 2], 5-aminolevulinic acid (5-ALA) fluorescence in malignant gliomas [3, 4], and intraoperative magnetic resonance imaging [5, 6], are employed to assist neurosurgeons in improving tumor resection, particularly in the infiltrative margins [7]. New procedures, including Raman spectroscopy (RS), hold promise for enhancing the goal of maximal tumor resection. RS, which is a label-free optical technique, provides intraoperative biochemical characterization of cancer tissue in situ [8, 9] and allows to distinguish brain tumors from normal brain parenchyma [10–12]. Over the past two decades, several studies have shown that the RS can not only identify neoplastic tissue of all types of brain tumors but also distinguish necrotic and infiltrative regions; moreover, it provides valuable information on tumor type, grade of malignancy and molecular markers [13–19].

When RS is applied to biological tissue, a significant portion of the acquired light signal originates from tissue autofluorescence (AF), which is mediated by cellular proteins and other fluorophores [20]. Traditionally, AF is considered a confounding factor and is either suppressed during the measurements or removed by data processing [21, 22]. Nevertheless, a few publications have shown that AF can provide useful information for distinguishing tumors from normal tissue [23–26]. In this study, we aimed to evaluate intraoperative spectroscopic detection of different brain tumors using a fiber-based commercial system. Our objectives were threefold: (i) to assess the reliability of in situ tumor detection by integrating spectroscopic analysis into the standard surgical workflow and minimizing measurement artifacts; (ii) to exploit the near-infrared AF that overlies the Raman spectra for identification of tumor tissue and analyze it in comparison to RS results; and (iii) to classify in vivo RS and AF datasets with a training set acquired ex vivo from resected tissue samples.

## Methods

### Patients

Measurements were performed during 29 routine brain surgeries for resection of different types of brain tumors. Ex vivo tissue samples were obtained from 44 brain tumor patients, and nonneoplastic brain tissue samples were obtained from four hippocampi resected during epilepsy surgeries (supporting Table S1).

### Raman probe and spectrometer

The measurement system consisted of a Raman spectrometer (type HT, EmVision, LCC, Loxahatchee, FL, USA) equipped with a cooled CCD camera (DU420A-BR-DD, Andor Technology Ltd., Belfast, UK), a fiber-based probe (EmVision, LCC, Loxahatchee, FL, USA), and a 785 nm diode laser (Innovative Photonic Solutions, Monmouth Junction, NJ, USA). The diameter of the irradiated region was 500 μm, and 95% of the Raman signal originated within a 1 mm depth [15]. The full technical specifications of the system are given in the Supporting Information.

### Raman probe sterilization and quality control

To ensure the use of the Raman probe in the operating room under sterile conditions, the probe was processed by the central sterile supply department (CSSD) of Dresden University Hospital. The processing consisted of two cleaning and sterilization steps. First, the probe was washed to eliminate any tissue or blood residues that would affect the outcome of the subsequent sterilization. For this purpose, a 15 min bath in 2% Gigasept AF Forte solution (Schülke & Mayr GmbH, Norderstedt, Germany) with a pH of 8.2 was established in accordance with standard internal protocols. The Raman probe was immersed in the bath up to the Y-piece, and the front window was then cleaned with ethanol to remove disinfectant residues. Sterilization of the probe was performed according to the manufacturer’s instructions using STERRAD plasma sterilization with a 100NX standard cycle.

To check whether the repeated cleaning and sterilization process damaged the Raman probe, the output laser power was measured, and the spectrum of an acetaminophen reference sample was recorded after 9, 21, and 31 cycles using the same instrument settings. The intensity of the acetaminophen Raman band at 858 cm^− 1^ remained constant until 21 cycles and decreased to 82% after 31 cycles. The measured output power decreased after 31 cycles to 84% of the initial value (i.e., from 81 mW to 69 mW). Correspondingly, some mechanical damage (scratches) to the front optical windows of the probe and to the surface of the excitation fiber were observed. This did not have a statistically significant effect on the acquired signal intensity over time (see Fig. S1 in Supporting Information).

### Intraoperative in situ spectroscopy

All persons in the laser hazard area were informed about laser safety and wore eye protection. Safety spectacles with interference-coated glasses were used, which enable transmission of visible light of 65% and excellent color recognition. The operating room was darkened, similar to the procedure for 5-ALA fluorescence microscopy. The swiveling LED lights above the operating table, the microscope and the neuronavigation system remained switched on. During routine tumor resection, the surgeon selected a suitable measurement position in the surgical area and placed the probe in contact with the tissue. First, a spectrum was acquired with the laser switched off to record the background of the room illumination. Subsequently, spectroscopic measurement of the tissue was performed with a nominal laser power of 100 mW, leading to an output power from the probe tip between 69 and 81 mW, depending on the probe age, as described in the previous section. Each spectrum was recorded with an exposure time of 0.05s and 40 accumulations, resulting in an acquisition time of 2s. Data that justify the choice of acquisition parameters are shown in Supporting Information and Fig. S3. In total, 218 spectra of 29 patients with brain tumors were acquired, both on the solid nonnecrotic tumor and on the tumor border (one spectrum per measurement point – supporting Table S1). In selected cases, a small tissue sample was collected from the region of interest for histopathological analysis.

### Ex vivo spectroscopy

Resected human tissue was transferred to an isotonic NaCl solution and analyzed within 1 h. A laser output power of 80 mW from the probe tip was used, while an enclosure enabled ambient light rejection. One to four spectra were acquired per sample, resulting in 375 spectra from 73 tissue samples of 48 brain tumor patients (supporting Table S1). Additionally, 108 spectra from two fresh unfixed mouse brains were recorded to develop the algorithm for artefact correction (see Supporting Information).

### Histopathology

Histopathological diagnosis was performed in the frame of routine patient care. Additionally, 80 tissue samples collected at selected measuring positions were stained for reference. The protocols used for histology and Ki67 immunohistochemistry are described in the Supporting Information.

### Spectral data processing and classification

The background spectrum was subtracted from the corresponding raw tissue spectrum. Afterwards, the obtained tissue spectra were processed in MATLAB 2021b (The MathWorks, Inc., Natick, MA, USA). AF and RS signals were separated with a baseline procedure, wherein the baseline curve represents the AF signal (supporting Fig. S2). Each curve was then subtracted from the corresponding tissue spectrum to retrieve the Raman spectrum. Finally, spectral artefacts were corrected in the Raman spectra, and the corrected spectra were vector-normalized.

The AF intensity was calculated as area under the curve (AUC) in the whole range 360–2040 cm^− 1^. The intensity of the lipid and of amide III Raman bands were calculated as sum of signals in the ranges (1437 ± 1.6) cm^− 1^ and (1260 ± 20) cm^− 1^, respectively.

The AF and RS datasets acquired in situ were classified using a model developed with the ex vivo dataset as the training set. The comparability of Raman spectra of ex vivo hippocampal tissue, in vivo brain tissue and ex vivo and in vivo tumors, is demonstrated in the Supporting Information and Fig. S4. Principal component analysis (PCA) was used to reduce dimensionality, and PCA coefficients were used as variables for classifying spectra as tumor or nonneoplastic with linear discriminant analysis. Patient-based K-fold leave-one-out cross-validation was used to estimate the classification performance on the training set. Classification of in situ datasets was performed using the first ten PCA coefficients for the RS dataset and the first three PCA coefficients for the AF dataset. The first ten Raman components explain 75.4% of the variability, higher components explain a very small variance and the corresponding eigenvectors only contain residual spectral artefacts and noise. The first three AF components explain almost 100% of the variance. Detailed descriptions of the data preprocessing, artefact correction, dimensionality reduction by PCA and classification procedures are provided in the Supporting Information.

### Statistics

Statistics were calculated using Prism 10 (GraphPad Software, Inc., San Diego, CA, USA). Different statistical methods were used and they are indicated in the Results section where appropriate. Differences were considered significant if *P* < 0.05.

## Results

**Fig. 1:**
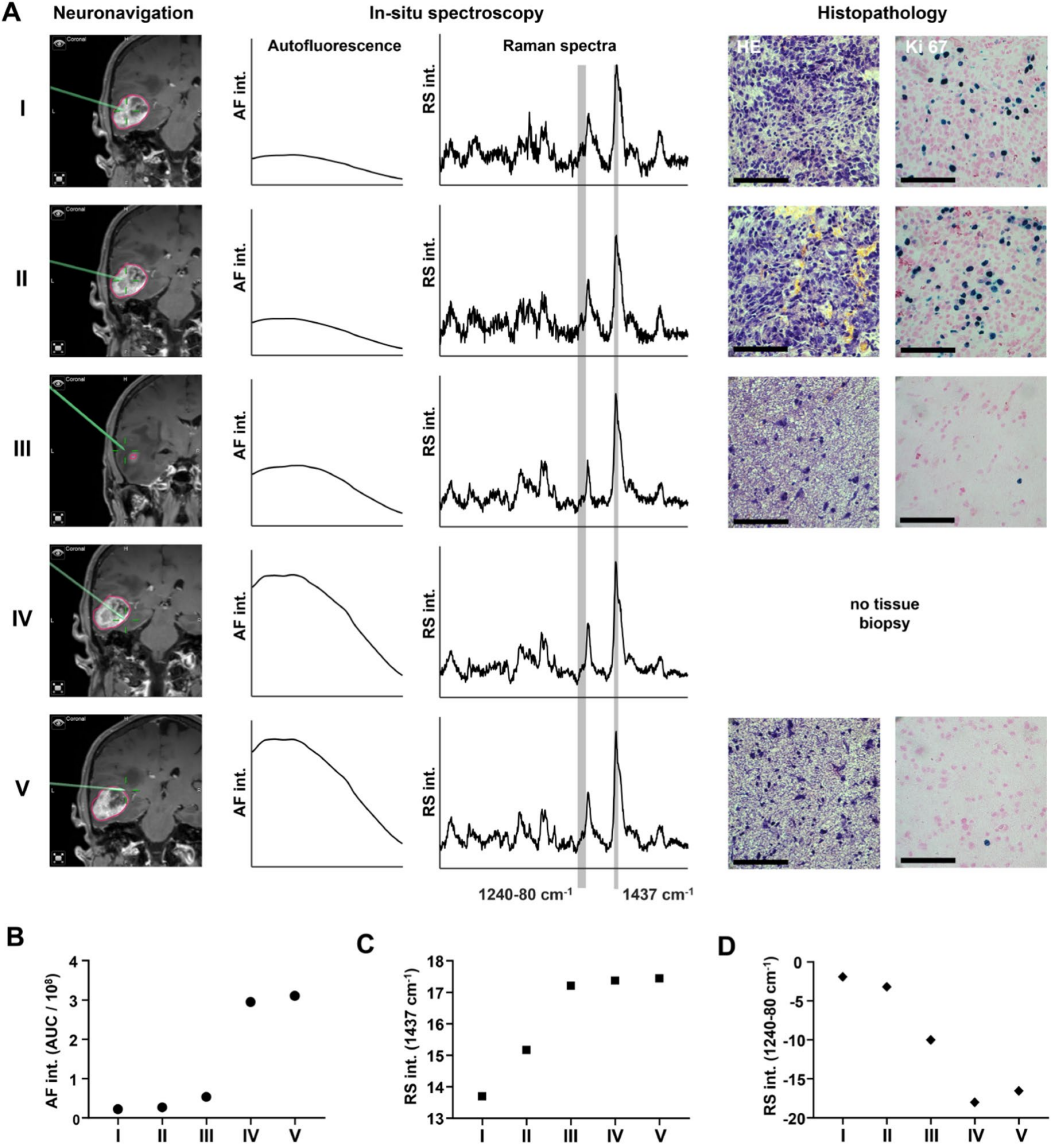
Intraoperative in situ spectroscopic exemplary measurement of a GBM. **A** Solid nonnecrotic tumor (positions I-II), tumor-infiltrated brain tissue (position III) and border regions with no or minimal infiltration (positions IV-V) were measured based on surgeon evaluation and neuronavigation. In situ spectroscopy was performed at these positions to retrieve autofluorescence (AF, here shown in the shift range of 360–2040 cm^− 1^), and Raman spectroscopy was also performed at these positions (RS AF, here shown in the shift range of 550–1800 cm^− 1^). Whenever possible (here for positions I, II, III and V), a tissue biopsy was excised at the measurement position and further processed for histology (HE) and immunohistochemistry (Ki67); all scale bars: 100 μm. **B** AF intensity. **C** Intensity of the RS band at 1437 cm^− 1^, representative of the lipid tissue content. **D** Intensity of the RS band in the range of 1240–1280 cm^− 1^, representative of the protein content

Based on the guidance of neuronavigation and the surgeon’s assessment, intraoperative spectroscopic measurements were conducted in situ on solid nonnecrotic tumors and at the tumor border, where very low or even no infiltration was expected. Figure 1 exemplifies this approach for a GBM patient and shows in situ AF and Raman spectra as well as retrospective reference immunohistochemistry for different positions of the tumor. The differences between solid tumors and border tissues are confirmed by the AF intensity, which is lower in neoplastic tissue than in normal tissue, as well as by Raman signal intensity in the spectral region 1240–1280 cm^− 1^ (amide III vibration of proteins [27]) and at 1437 cm^− 1^ (deformation vibration of CH_2_ groups in lipids [27]). These variations indicate increased protein content and corresponding decreased lipid content in the tumor.

As per the study protocol approved by the ethics commission, in situ spectral measurements were limited to the regions designated for resection. Therefore, intraoperative measurements of normal brain tissue distant from the tumor could not be obtained. To obtain reference spectra of nonneoplastic tissue, additional spectra were acquired from freshly resected samples of hippocampi from epileptic patients.

**Fig. 2:**
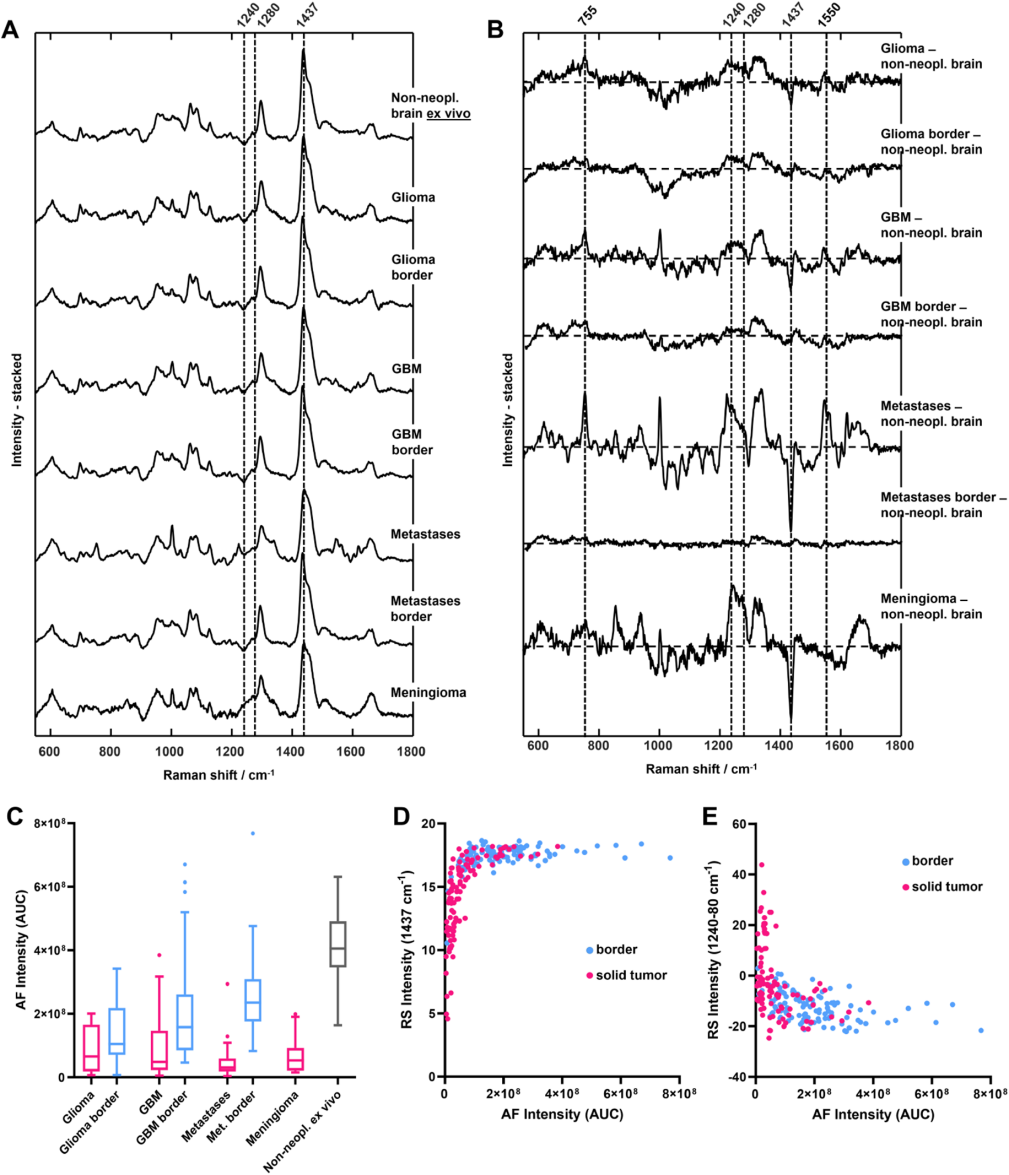
Intraoperative spectroscopy in situ. **A** Mean Raman spectra of tumor entities (solid tumor and tumor border) in comparison with the nonneoplastic brain ex vivo; border regions were not available for meningioma. **B** Mean difference spectra of tumors calculated using the mean spectrum of ex vivo nontumor tissue. **C** Median autofluorescence (AF) intensity (box and whiskers with Tukey method). **D** Scatter plot of AF intensity and intensity of the lipids at 1437 cm^− 1^ from Raman spectroscopy (RS). **E** Scatter plot of AF intensity and the intensity of the RS protein band between 1240 and 1280 cm^− 1^

Figure 2A shows the mean Raman spectra acquired in situ for all measured tumor types compared with the mean spectrum of normal brain tissue acquired ex vivo; the mean difference spectra are shown in Fig. 2B. These data show spectral differences underpinning the two main biochemical alterations that are common to tumors in the protein and lipid regions mentioned above. These spectral differences between solid tumors and nonneoplastic tissue become more pronounced with increasing malignancy in gliomas, while the largest changes are observed for metastases and meningioma. At the tumor border, all tumor entities exhibit spectrally similar yet smaller differences than solid tumors. These differences are more prominent for glioma WHO II-III and minimal in the case of metastasis borders, implying different degrees of infiltration in the border regions.

Despite the very high variability across measurements, the median AF intensity of all types of solid tumors (shown in Fig. 2C) is significantly lower than that of ex vivo nonneoplastic tissue (Kruskal‒Wallis test and Dunn’s multiple comparisons test, both *P* < 0.001). Furthermore, the AF intensity of tumor borders acquired in situ is higher than that of corresponding solid tumors for GBM and metastases (Dunn’s multiple comparisons test, *P* = 0.0017 and *P* < 0.001, respectively). Specifically, the difference is largest for metastatic lesions, which exhibit the lowest median AF intensity in the tumor tissue, and is not significant for gliomas WHO II-III and GBM. Interestingly, the RS and AF data are correlated, as shown by the scatter plots in Fig. 2D, E. The AF intensity exhibits a positive correlation with the intensity of the RS band of lipids at 1437 cm^− 1^ (Spearman’s *r* = 0.54, *P* < 0.001) and a negative correlation with the intensity of the protein band at 1240–1280 cm^− 1^ (Spearman’s *r* = -0.46, *P* < 0.001). The 3D scatter plot of AF intensity and both Raman bands is shown in Supporting Information, Fig. S7.

**Fig. 3:**
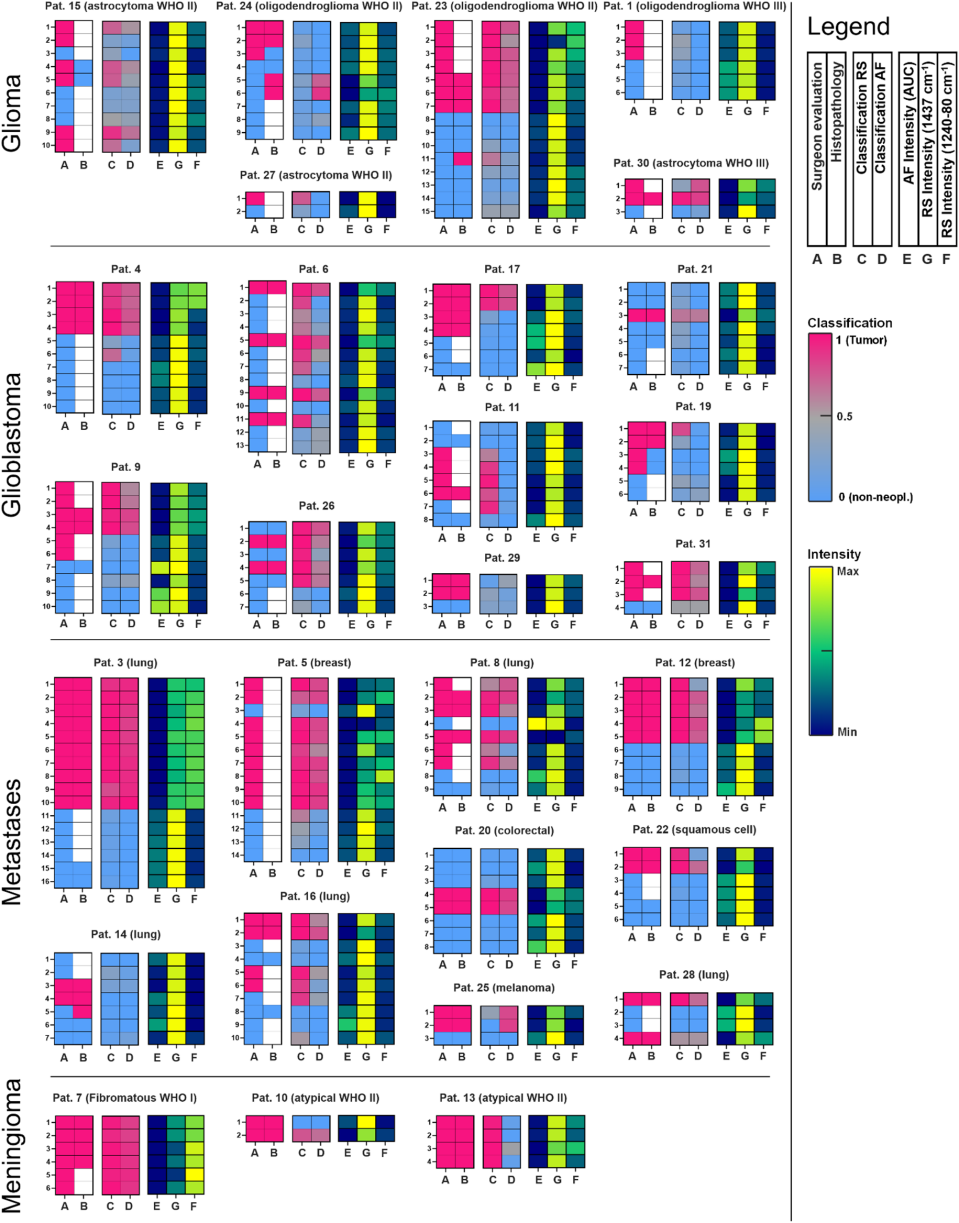
Classification of tumor tissue in situ. Each horizontal strip represents a spectrum; the ground truth, the classification probabilities, the AF intensity, and the intensities of the Raman bands at 1437 cm^− 1^ (lipids) and 1240–1280 cm^− 1^ (proteins) are coded as described in the legend. The patient number corresponds to the sterilization cycles already undergone by the Raman probe

To confirm the results of in situ measurements while avoiding artifacts from OR illumination and neuronavigation, ex vivo tumor tissue samples were also measured. Analysis of the RS and AF intensity data confirmed all the in situ measurements, including correlations between the AF and RS bands (supporting Fig. S6). Overall, the ex vivo measurements are in good agreement with previously published results obtained with laboratory equipment [24]. Moreover, there is a very good agreement between ex vivo and in vivo Raman spectra (see Supporting Information, Fig. S4). Therefore, the large ex vivo spectroscopic dataset (including spectra of nonneoplastic brain tissue excluding spectra of the dura) was used to train the classification models to be applied on intraoperative data. The results of classification of the intraoperative AF and RS datasets are shown in Fig. 3 and quantified in Table 1. The results of leave-one-patient-out classification of the ex vivo training set are shown for each spectrum and patient in Supporting Fig. S8. The AF intensity as well as the intensities of the RS band of lipids (at 1437 cm^− 1^) and proteins (at 1240–1280 cm^− 1^) are shown for additional reference.

The classification results of the RS data compared with the surgeon’s evaluation of tissue type at the measurement position showed that 79% of the spectra acquired from the tumor were classified as such, while 90% of the spectra acquired at the border were classified as nonneoplastic. The classification results of the subset of Raman spectra for which a biopsy was available were also compared with histopathological evaluation performed by an experienced neuropathologist. Eighty tissue biopsies provided direct histopathological reference information for 121 spectra, and the tissue was categorized by the pathologist as either neoplastic or as only slightly infiltrated (max 1–2% of tumor cells) or tumor free. The classification correctly recognized tumor for 80% of the Raman spectra of solid tumors and nonneoplastic tissue for 89% of the spectra of slightly infiltrated/nonneoplastic tissue. The classification results of AF spectra compared with surgeons’ evaluation of tissue type as well as histopathology provided comparable overall results. However, the sensitivity was rather low, as only 68% of the spectra were acquired from tumors classified as such.

**Table 1:** Classification results compared to the surgeon’s evaluation and histopathology (ground truth). The number of spectra is reported

	Surgeon’s evaluation		Classification RS		Classification AF	
	Tumor	Border	Tumor	Nonneopl.	Tumor	Nonneopl.
Glioma	22	23	14/22	23/23	12/22	22/23
GBM	34	41	25/34	32/41	19/34	39/41
Metastases	44	42	38/44	40/42	38/44	42/42
Meningioma	12	0	11/12	0/0	7/12	0/0
Total	112	106	88	95	76	103
Percentage			79%	90%	68%	97%
Total	218		183/218		179/218	
Percentage			84%		82%	

	Histopathology		Classification RS		Classification AF	
	Tumor	Infilt. 0–2%	Tumor	Nonneopl.	Tumor	Nonneopl.
Glioma	9	11	5/9	9/11	6/9	10/11
GBM	25	15	20/25	12/15	17/25	13/15
Metastases	31	20	26/31	20/20	26/31	20/20
Meningioma	10	0	9	0/0	5	0/0
Total	75	46	60/75	41/46	54/75	43/46
Percentage			80%	89%	72%	93%
Total	121		101/121		97/121	
Percentage			83%		80%	

For both methods, the best sensitivity was achieved for metastases (> 85%), which also displayed a pattern of classification consistent with the AF and RS intensities (for example, patient 3 in Fig. 3). The lowest sensitivity was obtained for glioma WHO II-III, which can generally be justified by the small spectral differences between tumor and ex vivo normal tissue highlighted in Fig. 2B. Furthermore, histopathology did not match surgeons’ evaluation for six samples of glioma and two samples of GBM (compare in Fig. 3); in these cases, it remains questionable which tissue was measured. Additionally, the AF and RS intensities are less consistent with tissue type for glioma and GBM (for example, see patients 15 and 9, respectively). A possible explanation is provided once again by the smaller differences between the tumor and border regions that can be extracted from the average spectra in Fig. 2A. In Fig. 3, the patient number gives the measurement order and corresponds to sterilization cycles that the Raman probe already underwent. No decline of classification performances was observed for the patients that were measured later in the course of the research, whereas interpatient and intertumoral variabilities dominate the classification rate.

**Fig. 4:**
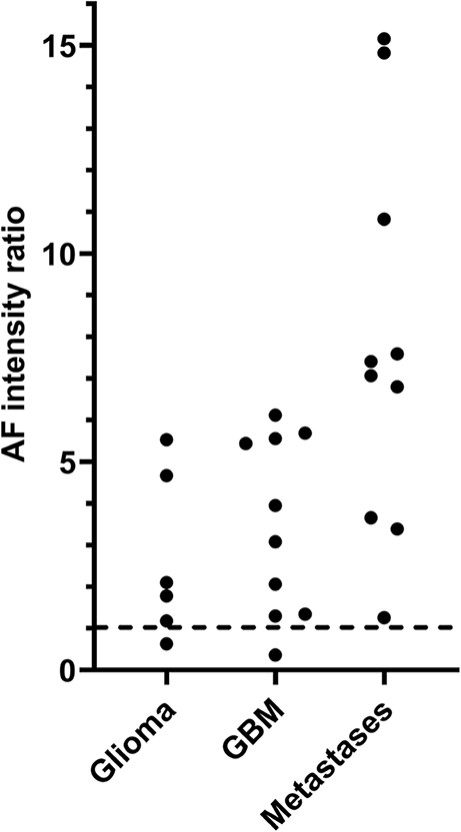
Autofluorescence intensity at tumor borders ratioed on the autofluorescence intensity of solid tumors. Each datapoint is the mean ratio for each patient

The lower classification performance provided by AF in comparison to that provided by RS is a consequence of the high interpatient variability in AF intensity (described by the PC n.1 and explaining the largest signal variance; see supporting Fig. S5b). This variability was found both in the tumor and in the border region; it was already suggested by the high scattering of the data in Fig. 2C and further confirmed by an interpatient analysis. However, the analysis of AF intensity in the tumor and in border regions of each patient showed that it was lower in the tumors of all patients, with only two exceptions (Fig. 4). The ratio between the AF intensity in the tumor region and that in the border regions is highly variable, but it is in most cases a reliable parameter for tumor identification irrespective of the tumor type.

## Discussion

In addition to the established techniques for the localization and visualization of brain tumors during surgery, the application of intraoperative in situ RS offers a valuable opportunity to enhance brain tumor removal and facilitate safe maximal resection. RS provides rapid biochemical information about the tissue under investigation, complementing established methods. Pioneering work on intraoperative RS was initially reported by Jermyn et al., who utilized a fiber probe for in situ detection of gliomas [15]. Subsequently, the same research group published studies highlighting technical advancements [28, 29], referenced tumor detection by RS using MRI data [14], and performed a multicenter study on GBM, metastases and meningioma [30]. Building upon these works, we successfully integrated spectroscopy into the surgical workflow without any complications. The medical staff demonstrated a high level of acceptance regarding the implementation of the essential laser safety regulations, which were promptly integrated into the standard operating procedures without any significant delays of surgeries. In our set-up, each measurement took 4s (2s for acquisition of the background and 2s for the spectrum), resulting in a minimal increase in surgical duration. Furthermore, we demonstrated that the fiber probe maintained sufficient optical performance after repeated sterilization, making it suitable for use in at least 30 operations. Larger variations in the transmitted laser power and/or collected signal intensity due to probe degradation during time can be compensated by periodic checks and consequent adjustment of the measurement integration time. This may further extend the lifetime of the fiber probe and renders the technique economically viable. We did not observe a systematic decline of classification results over time. If any is present, it is masked by interpatient variability.

The primary sources of interference in our study originated from the operating microscope light and the optical neuronavigation system. The correction of light and other spectral artefacts significantly hindering the interpretation of spectra is a well-recognized challenge [31]. Previous works by Jermyn and Desroches did not correct for these artefacts [15, 28, 29], while other groups emphasized the impact of preprocessing methods to allow direct comparisons among different RS studies on brain tissue [32]. We implemented background spectrum acquisition and subsequent subtraction techniques to eliminate external illumination signals. A further correction step eliminated all instrument-related artefacts, providing Raman spectra that allowed for accurate identification and quantification of bands. Although not essential for successful classification using artificial intelligence, this correction step is crucial for comprehending the underlying biochemical changes associated with classification, facilitating meaningful comparisons with other studies and enhancing the interpretability of the results.

Our findings confirm that RS is a reliable method for the intraoperative detection of gliomas, meningiomas and metastases [15, 30]. Therefore, we exploited RS as a reference for the interpretation of AF data.

AF spectroscopy and imaging in the visible range constitute well recognized tools for biomedical research and diagnosis in a variety of organs and enable delineating tumors. Interestingly, several neoplasms possess weaker AF compared to normal tissue [33, 34]. AF demonstrated especially useful in hepatology, as it enables to retrieve of a variety of diagnostic parameters, including energy metabolism, fibrosis and lipid accumulation in disease and cancer [35]. In normal liver and colorectal liver metastasis samples, visible and NIR AF was found significantly lower in the neoplastic tissue. Using excitation at 785 nm, a high level of tissue discrimination was achieved both on the basis of Raman spectra and AF intensity [36].

In the visible range, the majority of brain tumor types has significantly weaker AF compared to normal brain tissue [37], and the diagnostic potential of AF for the intraoperative delineation of GBM margins was already demonstrated [38]. Only few studies investigated brain AF in the near infrared upon excitation with long-wavelength light [24, 39]. It was shown different anatomical structures within the mouse brain have well-defined NIR AF fingerprints related to lipids and melanin [39], but detailed studies about the source of NIR AF in the human brain are not available yet. Our results confirm that AF intensity is generally lower in the tumors. We observed correlations between AF intensity and both lipid and protein content in the tissue retrieved by RS. However, we do not suggest lipids to be the source of AF but rather suggest an indirect relationship. In light of these results, we examined the AF intensity from the solid tumor to the border regions and found that its reduction in the tumor region was consistent within the same patient, although it can vary significantly from patient to patient. While the presence of interpatient variability limits the performance of classification models based on pooled datasets from multiple patients, our findings indicate that establishing a patient-specific threshold for AF intensity alone could delineate tumor borders.

Exploiting the near-infrared AF intensity would simplify the measurement process (both hardware and software) by eliminating the need for extensive spectral analysis while retaining the main advantages of long-wavelength excitation over standard visible fluorescence, such as increased safety against tissue photodamage. Intrapatient analysis also holds the potential to prolong the lifespan of fiber probes by complying with possible signal losses associated with fiber probe aging and repeated sterilization cycles.

The impossibility of generating systematic in vivo spectroscopic data from normal brain tissue is the limitation of this study, which hindered an evaluation of the ability of the system to differentiate between the normal brain and slightly infiltrated border regions. Therefore, further studies are required to investigate the sensitivity of AF for detecting infiltration. These should include measurements of tumor tissue, infiltrated tissue, and brain regions not foreseen for resection, i.e., tumor-free border tissue and distant normal brain parenchyma (which were lacking in the present study).

In summary, spectroscopic measurements can seamlessly integrate into established neurosurgical protocols, allowing for the classification of in vivo spectroscopic data based on ex vivo training datasets. RS has been confirmed to be effective at identifying brain tumors in their natural environment, while AF, which can complement RS or further develop as a stand-alone method, has emerged as an additional technique for delineating tumors intraoperatively.

## Electronic supplementary material

Below is the link to the electronic supplementary material.

Supplementary Material 1

## Data Availability

The datasets generated during and analysed during the current study are available from the corresponding author on reasonable request.
