# Supplementary material for: Raman and autofluorescence spectroscopy for in situ identification of neoplastic tissue during surgical treatment of brain tumors
Source: J Neurooncol. 2024 Aug 28;170(3):543–53. doi: 10.1007/s11060-024-04809-w (PMC11614956; doi:10.1007/s11060-024-04809-w)
Supplement: Supplementary file 1 — Supplementary Material 1 [file 11060_2024_4809_MOESM1_ESM.pdf]

Raman and AF spectroscopy for in situ identification of neoplastic tissue during surgical treatment of brain tumors

Ortrud Uckermann [1,2], Jonathan Ziegler [3], Matthias Meinhardt [4], Sven Richter [2,5], Gabriele Schackert [2], Ilker Y. Eyüpoglu [2], Mido M. Hijazi [2], Dietmar Krex [2], Tareq A. Juratli [2], Stephan B. Sobottka [2], Roberta Galli [3]

[1] Division of Medical Biology, Department of Psychiatry and Psychotherapy, Faculty of Medicine and University Hospital Carl Gustav Carus, Technische Universität Dresden, Dresden, Germany

[2] Department of Neurosurgery, Faculty of Medicine and University Hospital Carl Gustav Carus, Technische Universität Dresden, Dresden, Germany

[3] Medical Physics and Biomedical Engineering, Faculty of Medicine, Technische Universität Dresden, Dresden, Germany

[4] Department of Pathology (Neuropathology), Faculty of Medicine and University Hospital Carl Gustav Carus, Technische Universität Dresden, Dresden, Germany

[5] Else Kröner Fresenius Center for Digital Health, Faculty of Medicine, Technische Universität Dresden, Dresden, Germany

Co-first authorship: Ortrud Uckermann and Jonathan Ziegler

Corresponding author: Roberta.Galli@tu-dresden.de

Patients and acquired spectra

Table S1 Number of patients and acquired spectra.

**Ex vivo measurements**		
**Tissue type**	**n. of patients**	**n. of spectra**
Nonneoplastic	4	39
GBM	20	89
Metastases	11 ♦	52
Meningioma	13 †	195
Total	48	375

**In situ measurements**		
**Tumor type**	**n. of patients**	**n. of spectra**
Glioma WHO II-III	6 ‡	45
GBM	10	75
Metastases	10 ♣	86
Meningioma	3	12
Total	29	218

♦ Lung, n=4; breast, n=1; digestive tract, n=3; squamous cell carcinoma, n=2; melanoma, n=1

† Associated dura available for n=4 patients (n=41 spectra)

‡ Astrocytoma n = 3, oligodendroglioma n = 3

♣ Lung, n=5; breast, n=2; colorectal, n=1; squamous cell carcinoma, n=1; melanoma, n=1

Raman probe and spectrometer

The spectrometer and laser source are connected to the probe via a Y-piece using fiber optic fibers so that excitation and detection can be realized coaxially through the probe. For this purpose, the cylindrical tip of the probe, which has a diameter of 2.1 mm, is equipped with a two-component lens with a diameter of 2 mm at the front end. The probe is designed for immersion or direct contact measurements. The laser-guiding 300 µm-core low-hydroxyl (OH) fiber with a numerical aperture (NA)

of 0.22 is located in the center of the probe. A narrow bandpass filter centered on the laser wavelength is located at the front end. Seven identical 300 µm-core low-OH fibers (NA=0.22) surround the excitation fiber and collect the inelastic Raman scattering as well as the spectrally superimposed near-infrared autofluorescence (AF). A high-pass filter in front of the collecting fibers blocks elastic Rayleigh scattering.

The transmission holographic Raman spectrometer type HT from EmVision includes a spectrograph and a CCD camera (DU420A-BR-DD, Andor Technology Ltd., Belfast, UK) operated at a temperature of -60 °C. The system is equipped with an optical filter with an optical density > 6 at 785 nm to reject all residual elastically scattered light that was not filtered by the probe. The spectral range of the spectrometer is 350 - 2100 cm^{-1} (or 807 - 940 nm, given as the wavelength). The f-number is f/2.2, and the slit aperture size is 50 µm. The CCD camera is connected to a PC via a USB interface. Solis software (Andor Technology Ltd., Belfast, UK) was used to set the acquisition and to record the spectra. The spectrum of acetaminophen was used for system spectral calibration, which was performed following the manufacturer's protocol.

Histology and immunohistochemistry

All samples were fixed in 4% formaldehyde solution at 7 °C for 1–5 days and then were freeze-protected in ascending sucrose solution (10% and 30% for 24 h each). The samples were embedded in cryomedium, frozen on dry ice and then stored at -80 °C. Frozen tissue sections were prepared at a thickness of 10 µm and stored at -20 °C until staining.

Hematoxylin and eosin staining: The sections were washed in distilled water and incubated with Meyer’s hematoxylin/hemalum for 3 min. After washing in distilled water, the tissue was briefly destained in HCl-ethanol. Washing with tap water for 5 min was followed by 3 min of staining with eosin (1% eosin G in 80% ethanol). The sections were dehydrated in increasing ethanol concentrations, cleared in xylene and coverslipped with Entellan.

Ki67 staining: Tissue sections were allowed to thaw for 20 minutes at room temperature, fixed with a methanol-acetone solution at -20 °C, and then dried for 30 minutes. For heat-induced epitope recovery, cryosections were incubated in citrate buffer (pH=6.0) for 20 min in a steamer. Peroxidase blocking was performed with 0.3% hydrogen peroxide-methanol solution for 10 min. The block serum was added (0.3% Triton X-100 in 1% bovine serum albumin solution), followed by a 1 h incubation. The primary antibody (Novocastra monoclonal mouse antibody Ki67 antigen, Leica Biosystems) was then added to the blocking serum (1:200), and the sections were incubated for 1 h. After being washed with PBS, the sections were incubated with the secondary antibody (Histofine Simple Stain MAX PO (M), Nichierei Biosciences), followed by colorimetric detection of the antibody signal (HistoGreen Kit, Linaris). After the samples were washed again with PBS, counterstaining with nuclear red was performed. Finally, the sections were dehydrated in ethanol and xylene and coverslipped with Entellan. For each staining series, a positive control with histological reference material and a negative control without the addition of the primary antibody were used.

Signal intensity variation during the time course of the research

The Raman probe was subjected to sterilization procedure for each in vivo use and a gradual decrease in transmitted laser power was observed. However, no decrease in AF intensity could be detected over time during the research, as demonstrated by Fig. S1. It shows the AF intensity for each acquired

spectrum in the acquisition order. The slope of the linear regression is not significantly different from zero (P = 0.9863). This indicates that the interpatient variations in AF intensity are predominant over signal intensity variations due to Raman probe deterioration due sterilization and handling.

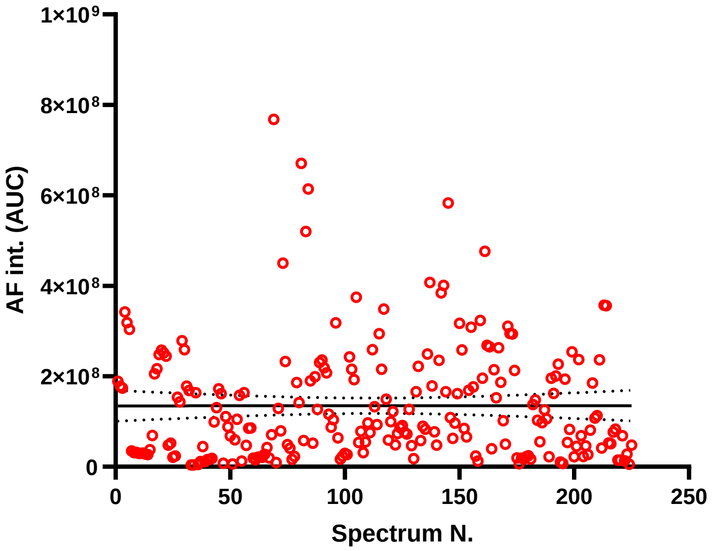

Fig. S1 Autofluorescence intensity in the time course of the research. The solid line is the linear regression; the dotted lines represent the 95% confidence interval.

Spectral preprocessing and correction of artefacts

After recording, the raw tissue spectra containing both the fluorescence signal and Raman spectrum, as well as the background spectra, were exported as ASCII files and further processed via the standard function in MATLAB 2021b (MathWorks, Inc., Natick, MA, USA).

First, the background was subtracted from the raw tissue spectrum, and then the obtained tissue spectra were reduced to the spectral range 366–2043 cm^{-1} to remove artefacts at the edges of the detection window (Fig. 2 a, solid lines). To separate the fluorescence and Raman signals, a baseline was calculated using the MATLAB function "msbackadj". For this purpose, a double linear interpolation was chosen as the regression method to determine the baseline with a window and step size of 100 cm^{-1} each. The baseline curve represents the fluorescence (Fig. S2 a, dashed lines). The baseline curve was then subtracted from the spectra to retrieve the Raman spectrum (Fig. S2 b, dotted lines). The spectral range was subsequently reduced to 472–2009 cm^{-1} to remove baseline artefacts at the spectral edges.

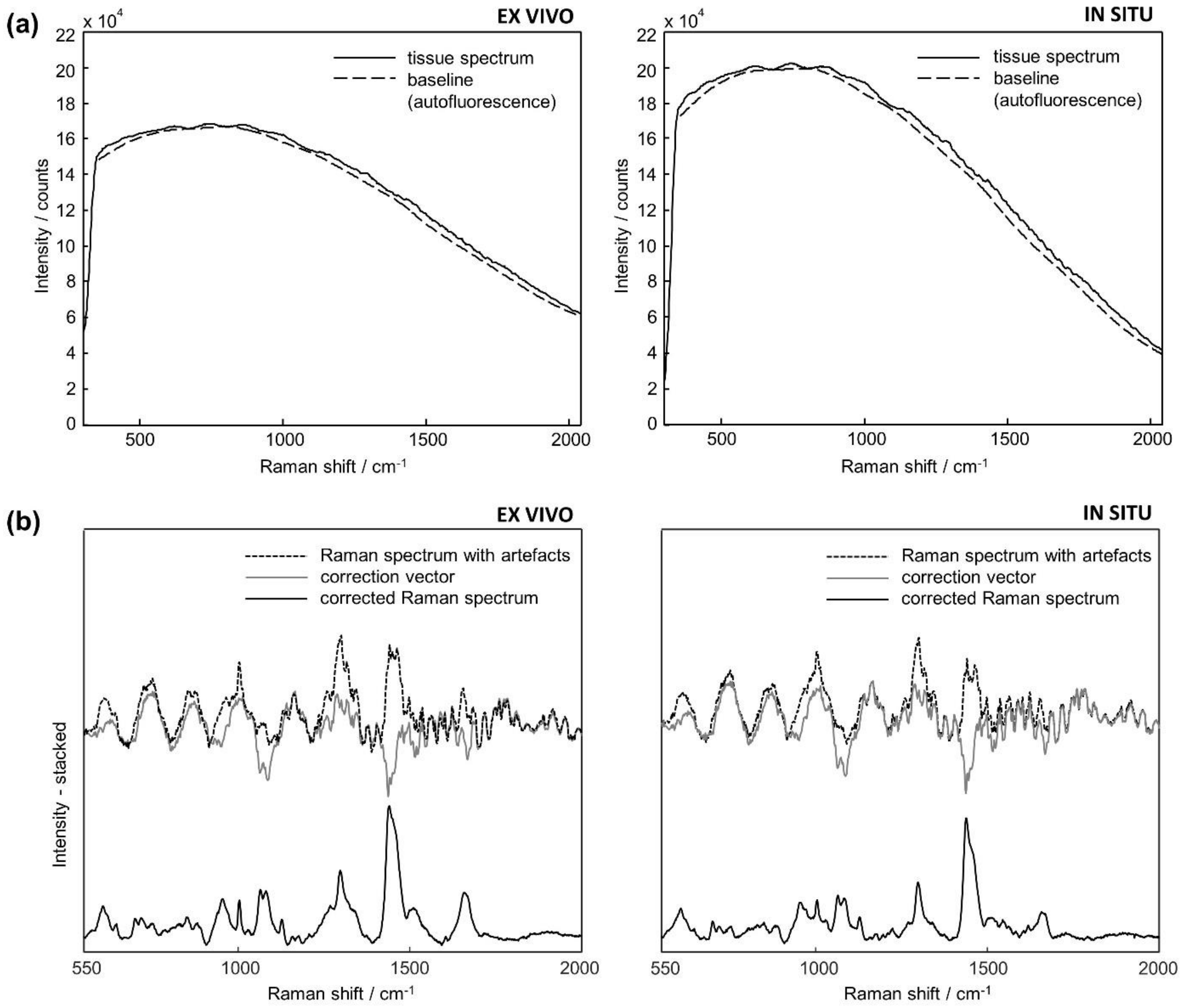

Fig. S2 Preprocessing of spectra. (a): Mean raw tissue spectra acquired ex vivo and in situ (solid line) and retrieved baseline (i.e., fluorescence) curve (dashed line) line. (b): Correction of Raman spectra for elimination of spectrometer artefacts: the mean Raman spectra of ex vivo and in situ measurements are shown before artefact correction (dotted black line) overlaid with the correction vector (gray line) that was multiplied by the mean value of the correction coefficient ($c_{\text{ex vivo}}$ = 1.27; $c_{\text{in situ}}$ = 1.44); the corrected Raman spectrum is the solid black line.

The Raman spectra obtained after preprocessing were affected by artefacts superimposed on the Raman bands. These artefacts depend on the amount of light entering the spectrometer and/or illuinating the CCD. In the case of brain samples, which are characterized by high fluorescence and weak Raman signals, the Raman bands are completely obscured. An algorithm for correction was then developed as follows.

A preliminary series of measurements was performed on 18 human and 2 fresh murine tissue samples, leading to the acquisition of a total of 444 spectra (108 spectra of the mouse brain, 112 spectra of the normal human brain from epilepsy surgery, and 224 spectra of human brain tumors). Principal component analysis ("pca" function of MATLAB) was applied to the spectra after preprocessing as described above. The second principal component fully described the artefacts (Fig. S2 b, gray lines), and the corresponding eigenvector (*v*) was used for correction. To adapt it to the individual artifact amplitude of each spectrum, a coefficient (*c*) was calculated for the spectral range devoid of Raman

bands between 1727 and 2009 cm^{-1} using the method of least squares so that the residual square sum between the respective uncorrected spectrum (y_0) and the correction vector was minimal. The difference in the spectrum and product of the coefficient and correction vector yields the artifact-corrected spectrum (y_1):

$$y_1 = y_0 - c \cdot v$$

The corrected Raman spectra (Fig. S2 b, solid lines) make it possible to interpret the band pattern. The spectra used to retrieve the correction vector are consistent with the spectra acquired ex vivo as well as in vivo; thus, this correction approach works well on all the data.

After correction, the Raman spectra were vector normalized before further analysis and classification were performed.

Determination of acquisition parameters

Three factors contributed to the determination of the acquisition parameters to be used in intraoperative measurements: i) avoid detector saturation, ii) assure good signal-to-noise ratio in Raman spectra by limiting detector shot-noise, iii) comply with surgical workflow.

A short integration time was necessary to keep the illumination of the detector always below saturation in the case of very intense tissue AF and limit the shot noise, which is proportional to the square of the detector illumination and thus increased by AF. On the other hand, a total acquisition time shorter than 5 s was desired by the surgeons. Based on these indications, the analysis of the best acquisition parameters was performed on different brain and tumor samples ex vivo before starting the research.

Therefore, the signal quality was evaluated in ex vivo measurements for total acquisition times between 0.5 and 4.0 s for different integration times and numbers of accumulations. Two seconds of acquisition was judged as the best compromise between noise and duration. Within this acquisition time, differences in noise due to integration time are negligible; however, we still rarely observed detector saturation for an accumulation of 0.1 s. Therefore, an integration time of 0.05 s and 40 accumulations were selected for intraoperative measurements.

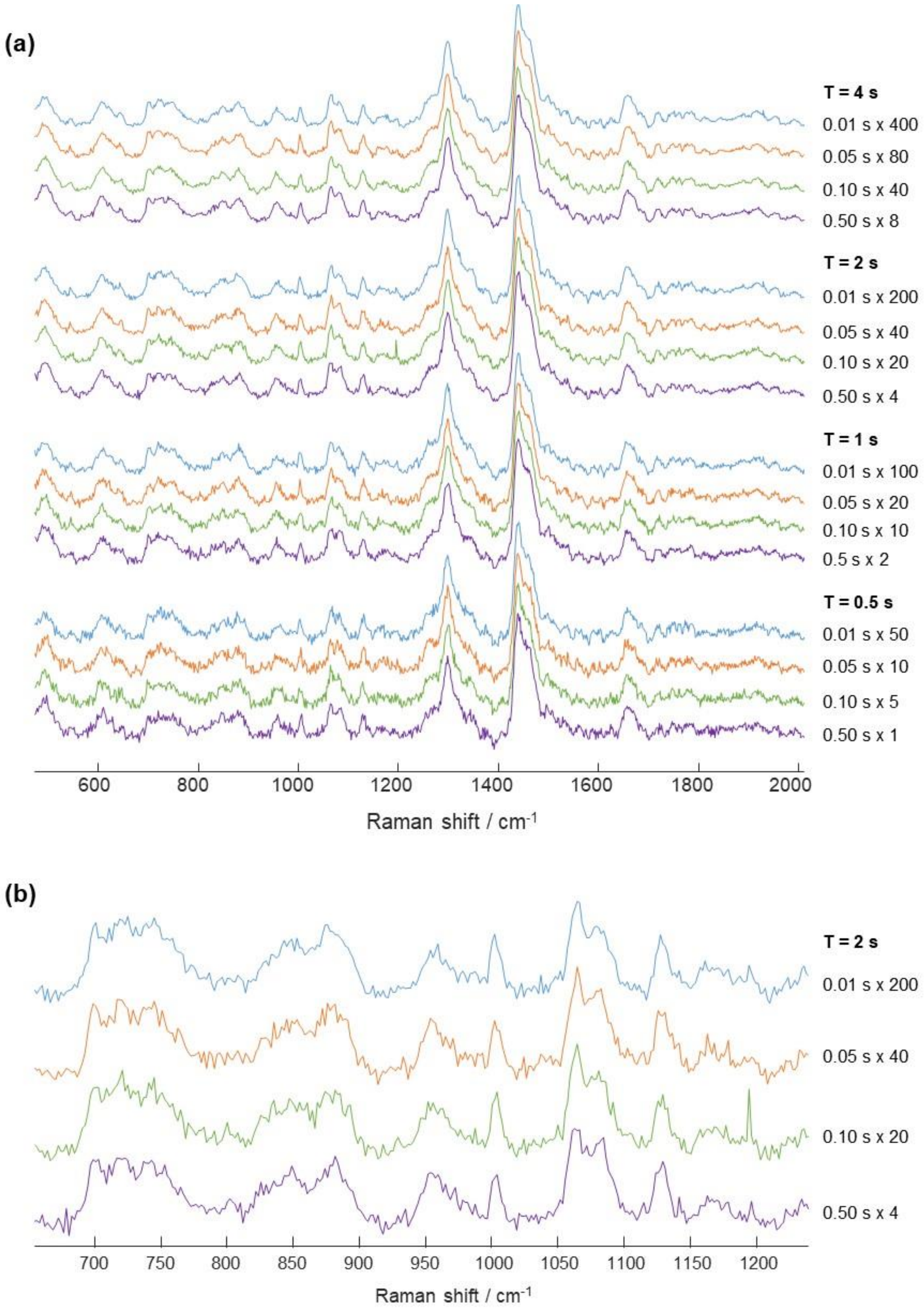

Fig. S3 Effects of acquisition parameters on the quality of Raman spectra. (a) Raman spectra acquired with different combination of integration times and accumulation numbers, leading to total acquisition times T between 0.5 and 4 s. (b) Magnification of spectra for the selected total acquisition time T = 2 s shown in a spectral region with weak Raman bands. Raman spectra of gray matter ex vivo are shown after baseline procedure, artefact correction, and normalization.

Comparison of Raman spectra of ex vivo and in vivo brain tissue and tumor tissue

The comparability of Raman spectra acquired ex vivo and in vivo was analyzed in order to define feasibility of classification of in vivo datasets based on algorithms trained on ex vivo datasets. Figure S4a shows the comparison of mean Raman spectra of GBM acquired ex vivo and in situ, together with the difference spectrum. Despite the expected interpatient variability and the limited number of spectra and patient used for this analysis, the mean spectra are very similar and no bands appear in the difference spectrum. Figure S4b shows the comparison of mean Raman spectra of hippocampal tissue acquired ex vivo together with the mean Raman spectra of a sub-group of spectra acquired in border regions of gray and white matter that were expected to be only slight infiltrated or tumor-free based on surgeon indications. The spectra of hippocampal tissue and of gray matter are very similar, as also confirmed by absence of bands in the difference spectrum.

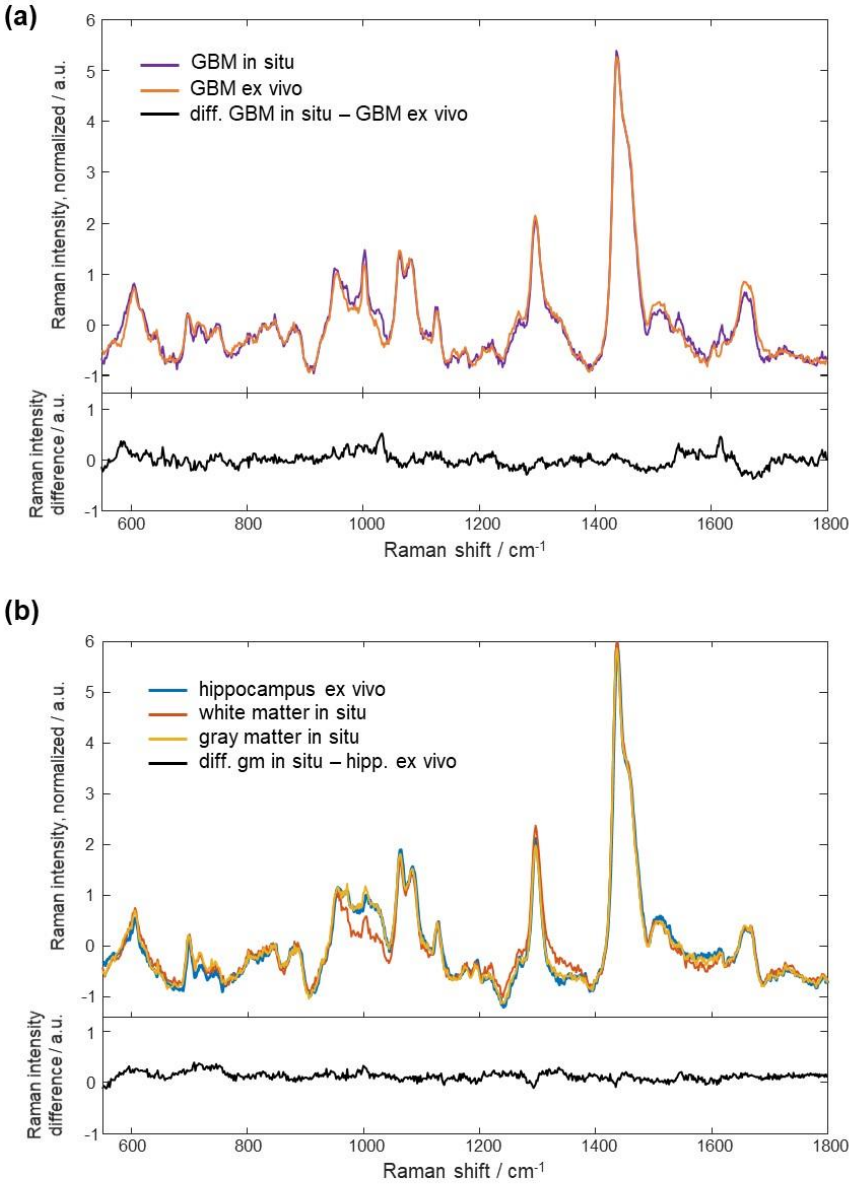

Fig. S4 Comparison of Raman spectra acquired ex vivo and in situ. (a) Mean Raman spectra of GBMs acquired in situ (34 spectra) and ex vivo (83 spectra), and corresponding difference spectrum. (b) Mean Raman spectra of hippocampal tissue and in situ measurements of border regions of gray and white matter (hippocampus ex vivo: 39 spectra, gray matter in situ: 22 spectra, white matter in situ: 18 spectra).

Dimensionality reduction with principal component analysis and development of the classification model

Only Raman and AF spectra of normal brain tissue and of tumors measured ex vivo (i.e., excluding spectra of the dura) were used to build the training set for classification. Principal component analysis (PCA) of the Raman and fluorescence spectra of ex vivo measurements was performed using the MATLAB function "pca". The eigenvectors of the principal components used for classification are shown in Fig. S5. The PCA coefficients of the Raman and AF datasets were handed over to discriminant analysis for classification into two groups, i.e., normal tissue and neoplastic tissue. The discriminant analysis was performed with the MATLAB function "classify". Leave-one-out cross validation was used to define the type of discriminant function (i.e., linear or quadratic) and the number of principal components to be used to maximize classification performance. For instance, K-fold leave-one-out cross-validation was used for estimation of performance, with K equal to the number of patients (i.e., leave-one-patient-out cross-validation).

For the Raman data, a cross-validation correct rate of 0.8623 (sensitivity = 0.8475, specificity = 0.9744) was achieved using the first ten coefficients and linear discriminant analysis. The first ten coefficients explain 75.4% of the variability, higher component explain a very small variance and the corresponding eigenvectors only contain residual spectral artefacts and noise. For AF data, the best classification in the cross-validation was achieved again with linear discriminant analysis but using only the first three PCA coefficients, which explain almost 100% of the variance. The correct rate of cross-validation was 0.9251, with sensitivity = 0.9322 and specificity = 0.8718. The classification results of the cross-validation of the AF and Raman training datasets are described in Supporting Fig. S8. The dataset acquired in situ was classified using ex vivo data as the training set. Therefore, the PCA coefficients of the in situ Raman and fluorescence datasets were retrieved on the basis of eigenvectors of the corresponding ex vivo datasets and then classified with the model developed on the ex vivo datasets.

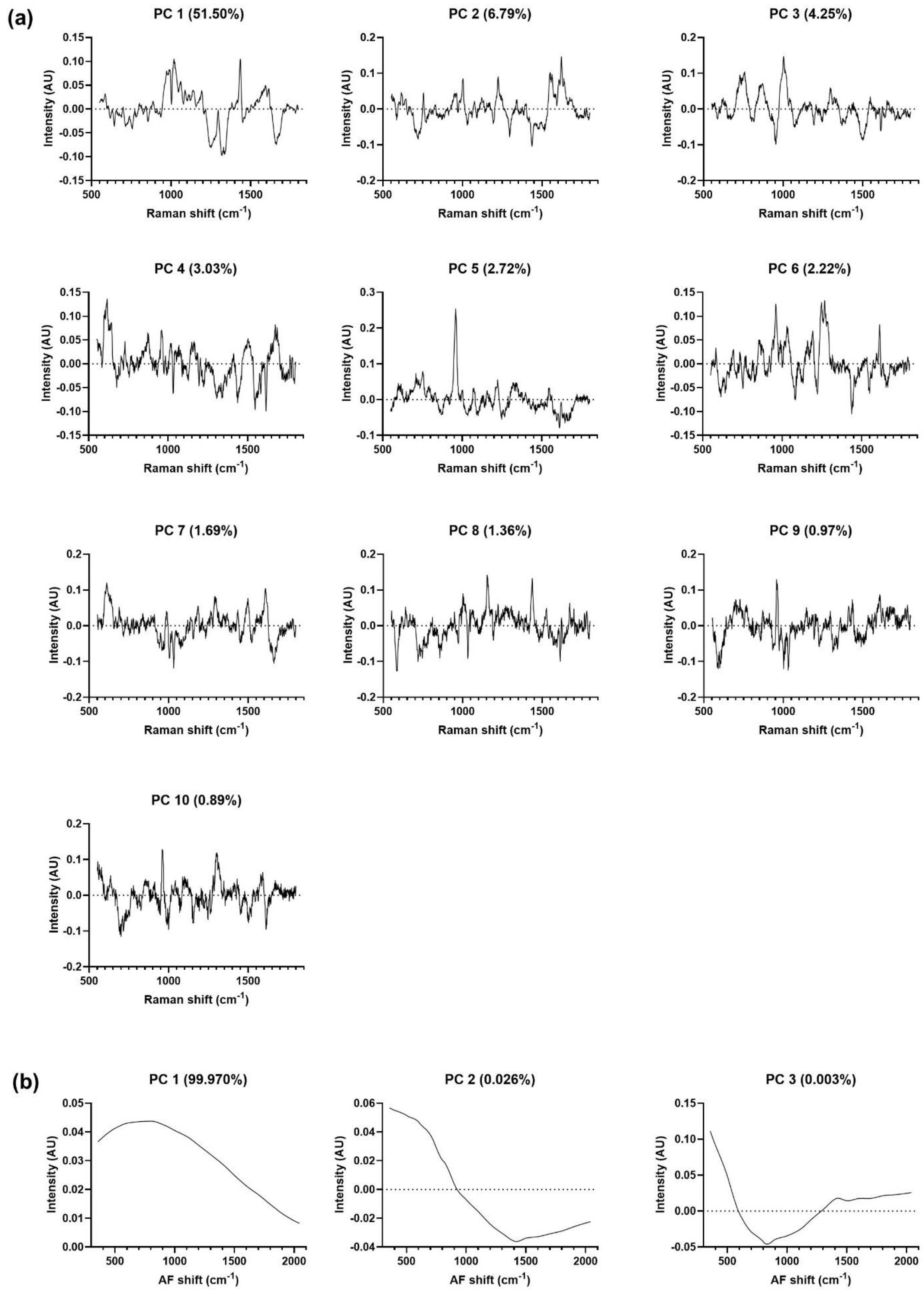

Fig. S5 Principal component analysis. (a): Eigenvectors of the first ten components of the Raman dataset; (b): eigenvectors of the first three components of the AF dataset. The percentage given in parenthesis is the variance explained by each component.

Spectroscopy of ex vivo human samples

Spectra were acquired from freshly resected samples of brain tumors and hippocampi from epileptic patients. The mean Raman spectra (retrieved after preprocessing and spectrometer artefact correction of each single spectrum, Fig. S6a show differences among tissue types. Spectral variations in the overlapping bands can be observed in the difference spectra, which are obtained by subtracting the mean Raman spectrum of the nonneoplastic brain from the mean Raman spectra of each tumor type (Fig. S6b). These differences are represented by the protein band at 1240–1280 cm^{-1} (amide III vibration) and the lipid band at 1437 cm^{-1} (deformation vibration of CH_2 groups). The dura contains more proteins and less lipids than both brain tissue and meningioma. Therefore, the Raman spectrum of meningioma is different from that of nonneoplastic brain tissue as well as from the dura from which it originates.

The AF intensity is shown in Fig. S6c. The median AF intensity of nonneoplastic brain tissue was greater than that of all tumor types and the dura, and significant differences existed among the medians of the five tissue types (Kruskal–Wallis test, $P < 0.001$). However, the scattering of single values, as well as the overlap among tissue types, is high.

The information carried by the Raman spectra and AF intensity are linked (Fig. S6 d, e). The AF intensity is positively correlated with the intensity of the Raman band at 1437 cm^{-1} (Spearman $r = 0.86$, $P < 0.001$) and negatively correlated with the intensity of the amide III spectral feature at 1240-1280 cm^{-1} (Spearman $r = -0.84$, $P < 0.001$).

The tridimensional scatter plot of AF intensity vs. lipid and protein Raman bands are additionally shown below to show the clustering of data (Fig. S7).

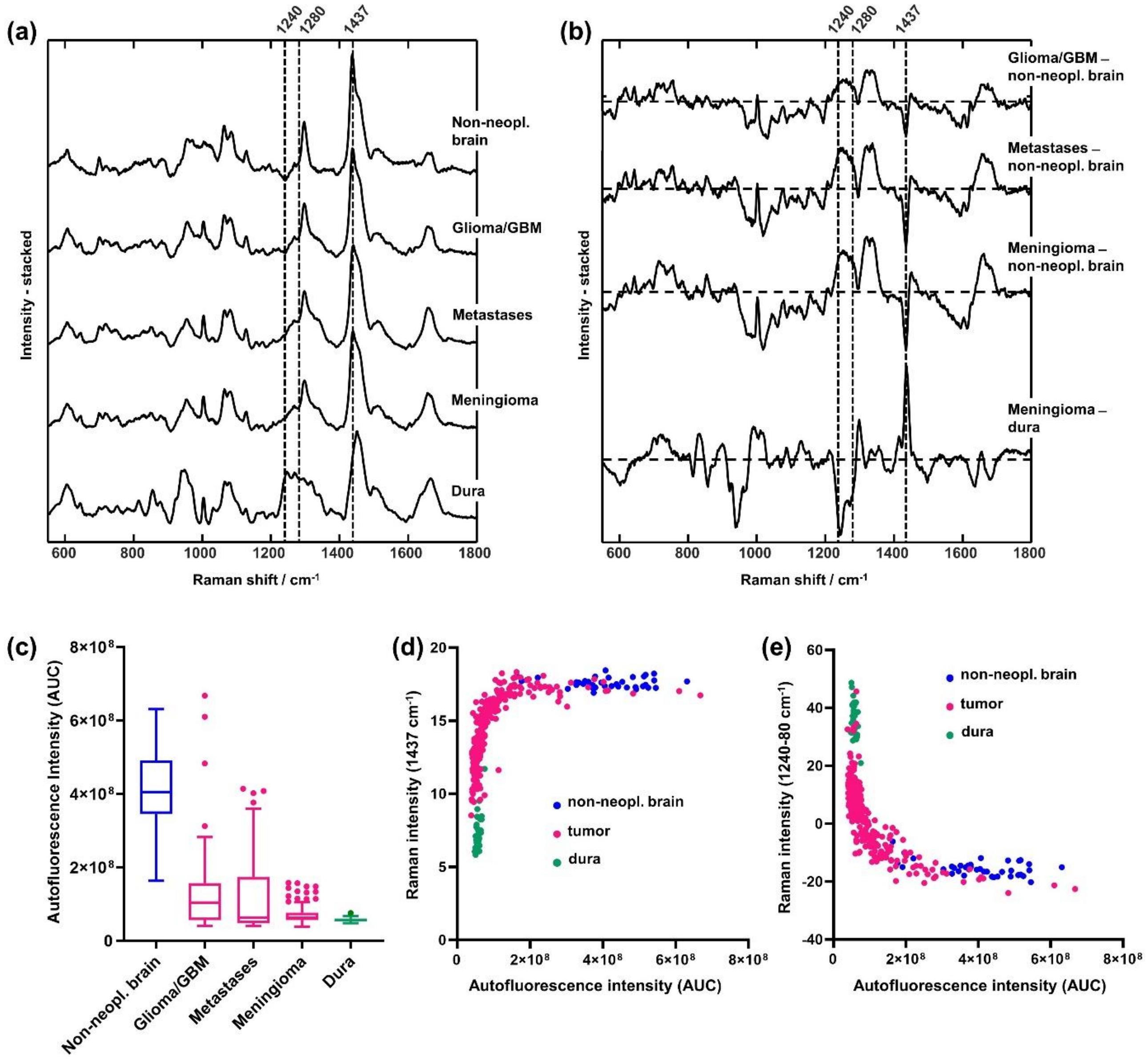

Fig. S6 Spectroscopy of ex vivo tissue samples. (a): Mean Raman spectra. (b): Mean difference spectra of tumors and nonneoplastic brain tissue. (c): AF intensity; the value of each measurement is reported as the median for each group. (d): Scatter plot of the AF intensity and intensity of the Raman band of lipids at 1437 cm^{-1}. (e): Scatter plot of the AF intensity and intensity of the amide III protein Raman band between 1240 and 1280 cm^{-1}.

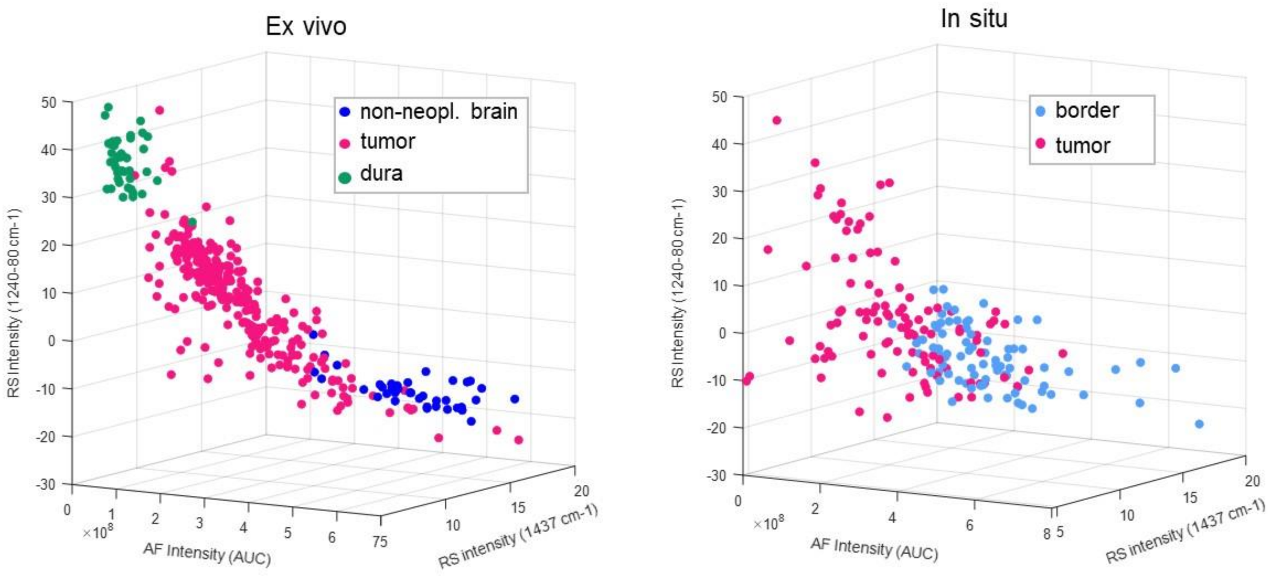

Fig. S7 Tridimensional scatter plots of AF and Raman bands intensity. These diagrams resume Fig. 2 panels d and e of in vivo data, and supporting Fig. S6 panels d and e of ex vivo data.

Classification of ex vivo data

The classification results from the cross-validation of the Raman and AF training sets are shown for each spectrum and patient in Fig. S8. The intensities of AF, as well as those of the lipid and protein Raman bands (1437 cm^{-1} and 1240-80 cm^{-1}, respectively), are given as references to help interpret the classification results.

A comparison of reclassifications based on Raman spectra and AF revealed that Raman spectroscopy is best for the recognition of nonneoplastic brain tissue, whereas AF provides better results, especially for the recognition of gliomas and glioblastomas. However, samples with many misclassified spectra were incorrectly classified by both approaches (e.g., nonneoplastic samples from Patient 1, glioma/GBM samples from Patients 16 and 19, and metastatic samples from Patients 1 and 11). Furthermore, spectra that are incorrectly classified on the basis of Raman data typically display an intensity of lipid and amide III bands that are similar to those of nonneoplastic tissue, whereas spectra that are misclassified on the basis of AF display high AF intensity similar to that of normal tissue. Notably, several tumor spectra with high lipid contents and low protein band intensities were nevertheless correctly classified; this is explained by the fact that several principle components were used for classification, which consequently resulted from the use of more spectral features reflecting additional biochemical alterations.

Samples of GBM (patients 10, 13, 16 and 19) and metastases (patients 1 and 11) were more often wrongly classified. Retrospective histopathological evaluation of the tissue of these samples revealed that, in two patients, the GBM tissue was necrotic (patients 13 and 16), whereas in two patients, a border region with normal tissue or with very few tumor cells was observed (patients 10 and 19). For metastases, sample n.1 was a border region of normal tissue, whereas sample n.11 was not assessable.

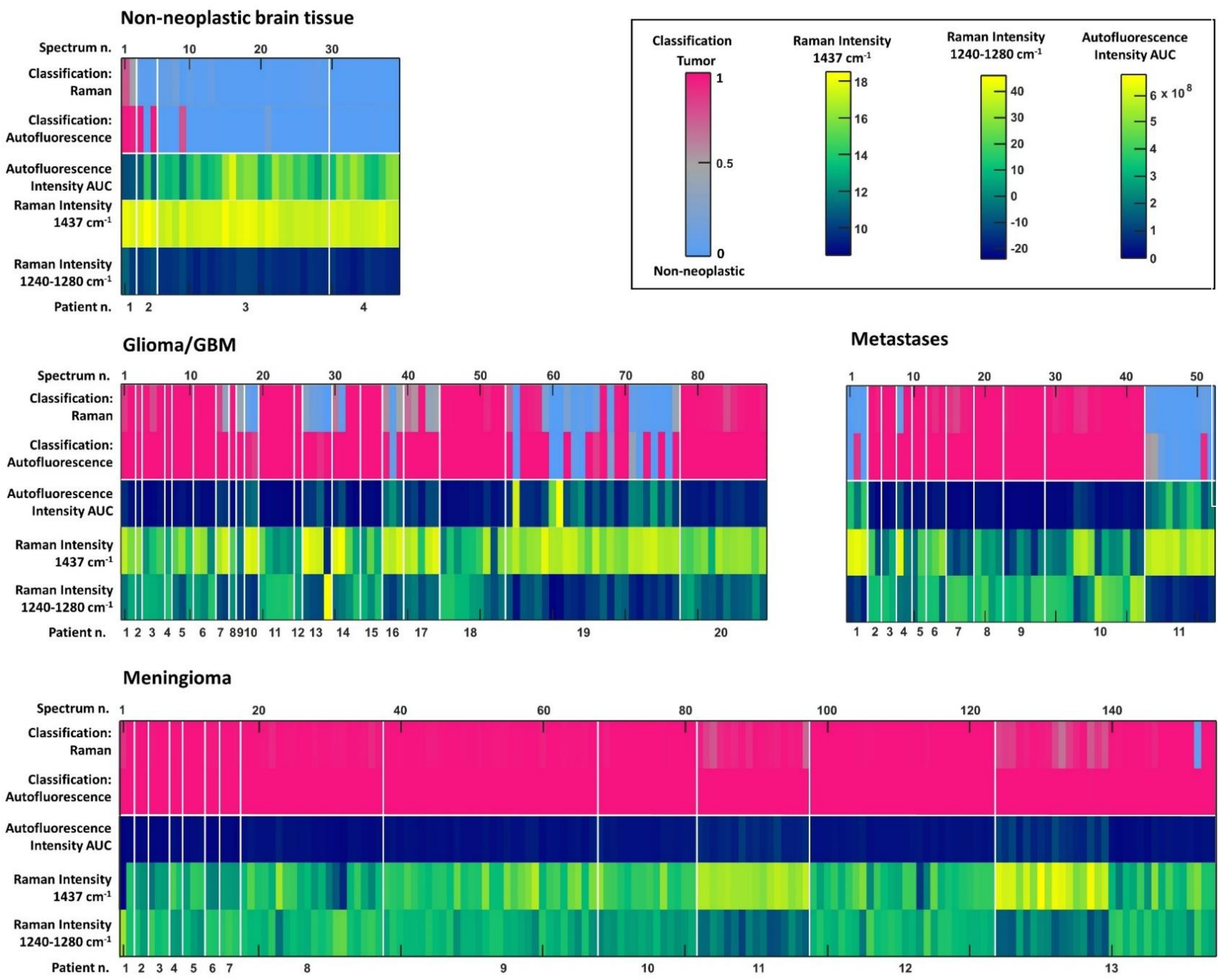

Fig. S8 Reclassification of ex vivo tissue samples. Reclassification results (training set) are shown for each spectrum of each patient split for tissue type. Each vertical strip represents a spectrum and the color codes classification results as well as intensities of Raman bands at 1437 cm^{-1} (lipids) and 1240-1280 cm^{-1} (amide III) and AF intensity. White lines separate groups of spectra of each patients.
